# Non-Operating Room Anesthesia (NORA) for Ultrasound-Guided Liver Radiofrequency Ablation

**DOI:** 10.3390/diagnostics14161783

**Published:** 2024-08-15

**Authors:** Carlo Felix Maria Jung, Elisa Liverani, Cecilia Binda, Ludovica Cristofaro, Alberto Gori, Luigina Vanessa Alemanni, Alessandro Sartini, Chiara Coluccio, Giulia Gibiino, Chiara Petraroli, Carla Serra, Carlo Fabbri

**Affiliations:** 1 Gastroenterology and Digestive Endoscopy Unit, Forlì–Cesena Hospitals, AUSL Romagna, 47121 Forlì, Italy; elisa.liverani@auslromagna.it (E.L.); cecilia.binda@auslromagna.it (C.B.); vanessaalemanni1@gmail.com (L.V.A.); alessandro.sartini@auslromagna.it (A.S.); chiara.coluccio@auslromagna.it (C.C.); giulia.gibiino@auslromagna.it (G.G.); chiara.petraroli@auslromagna.it (C.P.); carlo.fabbri@auslromagna.it (C.F.); 2 Department of Medical and Surgical Sciences—DIMEC, Alma Mater Studiorum–University of Bologna, 40138 Bologna, Italy; cristofaroludovica@outlook.it; 3 Anesthesiology and Intensive Care Unit, Morgagni Pierantoni Hospital, AUSL Romagna, 47121 Forlì, Italy; alberto.gori@auslromagna.it; 4 Interventional, Diagnostic and Therapeutic Ultrasound Unit, IRCCS, Azienda Ospedaliero—Universitaria di Bologna, 40138 Bologna, Italy; carla.serra@aosp.bo.it

**Keywords:** non-operating room anesthesia (NORA), deep sedation, percutaneous liver radiofrequency ablation, hepatocellular carcinoma

## Abstract

Introduction: Percutaneous ultrasound-guided radiofrequency ablation (RFA) is a well-studied treatment option for locally non-advanced hepatocellular carcinoma (HCC) and colorectal liver metastases (CRLMs). Sedation is of crucial interest as it enables safe and pain-free procedures. Whether the type of sedation has an impact on procedural outcome is still not well investigated. Methods: We retrospectively collected data on patients undergoing liver RFA for various oncological conditions. Procedures were conducted in a non-operating room anesthesia (NORA) setting. Procedural-related complications and short-term oncological outcomes were analyzed. Results: Thirty-five patients (mean age 71.5 y, 80% male) were treated for HCC (26), CRLM (6) and gastric cancer metastases (3). Mean lesion size was 21 mm (SD ± 10.1 mm), and the most common tumor localization was the right hepatic lobe. RFA was performed in a step-up sedation approach, with subcutaneous lidocaine injection prior to needle placement and subsequent deep sedation during ablation. No anesthesia-related early or late complications occurred. One patient presented with pleural effusion due to a large ablation zone and was treated conservatively. Local tumor-free survival after 1 and 6 months was 100% in all cases where a curative RFA approach was intended. Conclusions: NORA for liver RFA comes with high patient acceptance and tolerance, and optimal postoperative outcomes and oncologic results.

## 1. Introduction

Percutaneous liver radiofrequency ablation is an established and well-studied local treatment method for hepatocellular carcinomas (HCCs) and colorectal liver metastases (CRLMs). In addition to radiofrequency ablation (RFA), several local ultrasound-guided treatments are to date available, namely microwave ablation, cryoablation and laser ablation [1,2]. The Barcelona Clinic Liver Cancer (BCLC) staging system proposes local ablation methods for up to three HCC nodules and a maximum dimension of 3 cm; the European Society for Medical Oncology (ESMO) guidelines consider local ablation methods for liver metastasis up to 3 cm [3,4]. The aim of these ablation techniques is to obtain complete tumor necrosis, creating a safety margin around the treated lesions. RFA, microwave ablation (MWA), high-intensity focused ultrasound and laser therapy are hyperthermic procedures that apply energy to tissue to at least 60 °C for maximum efficacy. Cryoablation instead is a hypothermic procedure, leading to tissue necrosis by cooling to less than −40 °C. The effectiveness of treatment depends on numerous factors, such as tumor size, tumor location, the proximity to large vessels and the equipment utilized.

Recently, MWA has gained popularity as it may produce larger ablation zones in a shorter procedure time and is not altered by the heat sink effect. Therefore, ablation zones are easier to predict. Larger randomized trials to date have failed to demonstrate a clear advantage of MWA compared with RFA [5,6,7].

Thermoablation treatments have also been described for tumor debulking in lesions larger than 3 cm, in combination with transarterial chemoembolization [8,9]. Ablations can be performed under computed tomography (CT scan) or ultrasound guidance. Ultrasound-guided interventions have important advantages compared with CT scans, including better maneuverability under constant vision, a shorter procedure time, no use of radiation and lower costs. Oncological outcome is not different [10,11,12].

RFA has demonstrated valuable results over the last few years. Indeed, local recurrence-free survival after 5 years is 13–40% after RFA for HCC. Recurrence rates after RFA for CRLM after 2 years are up to 13% [13,14,15]. RFA therefore is a valuable alternative to surgical resection in selected patients, although larger trials comparing surgery with RFA are not available yet.

The procedure can be performed using local anesthesia, conscious or deep sedation, or general anesthesia [10,11,12,16]. However, how the type of sedation during the procedure may affect procedural outcomes has not yet been largely studied. To the best of our knowledge, few retrospective studies and only one randomized trial exist discussing this issue, having a focus on post-interventional outcomes, such as hospitalization time and postoperative complications [17,18,19,20]. The application of deep sedation protocols requires experienced anesthesiologists, especially in patients with multiple comorbidities. Conscious sedation might be difficult in patients with a history of alcohol or drug abuse, as preexisting adaptation to sedatives may be present. Moreover, patients with metabolic liver disease often have multiple cardiometabolic comorbidities including obesity.

Airway management is especially crucial in these patients, who may experience nausea and vomiting during thermoablation as a common side effect. Physicians trained in airway protection are therefore crucial for these procedures.

The aim of this study is to describe the role of deep sedation in a non-operating room anesthesia (NORA) setting for percutaneous liver radiofrequency ablation for HCC, CRLM and liver metastases from gastric cancer, in order to evaluate any potential advantages and disadvantages of this approach.

## 2. Materials and Methods

We retrospectively analyzed all consecutive radiofrequency ablations performed in our Gastroenterology Unit (Department of Gastroenterology and Digestive Endoscopy, Forli Hospital AUSL Romagna, Italy) between September 2021 and October 2023. Main indications were hepatocellular carcinoma, CRLM and liver metastases from gastric adenocarcinoma. All cases were discussed by our multidisciplinary tumor board, and indications adhered to current BCLC and ESMO guidelines.

Inclusion criteria for HCC for RFA treatment were a very early and early stage HCC/ECOG performance score 0, preserved liver function and up to 3 nodules with a size ≤ 3 cm. The same criteria applied for patients presenting with colorectal liver metastases unfit for surgery or whenever surgically non-approachable. Written informed consent was acquired for all patients.

Exclusion criteria were age less than 18 years, patients unable to provide adequate informed consent, hepatic lesions non-approachable or visible by ultrasound, severe and non-correctable coagulopathy, lesions adjacent to the gallbladder with signs of infiltration and lesions with a localization < 5 mm to bile duct or major vascular structures. Patients with ascites, poor ECOG performance status and CHILD B/C were excluded.

In patients receiving anticoagulants, adequate suspension and bridging with low-molecular-weight heparin subcutaneously was performed whenever necessary. In patients with antiaggregant treatment, including clopidogrel, ticagrelor or prasugrel, antiaggregant treatment was suspended at least 5 days prior to the procedure. If not possible, a switch to acetylsalicylic acid was performed and continued throughout the procedure.

### 2.1. The Technique

Thermoablation of liver tumors is generally performed in specialized rooms, providing enough space for equipment and staff. This includes the operator, dedicated nurses, and, if performed in a NORA setting, anesthesiologists and anesthesia nurses, a respirator—and ultrasound equipment.

Thermoablations with RFA were performed with an ultrasound-guided technique, using a Logiq E10 Series Ultrasound system (GE Healthcare, Chicago, IL, USA) and a dedicated needle guidance system (VERZA, CIVCO Medical Solutions, Kalona, IA, USA). All procedures were conducted in a NORA setting, under deep sedation using intravenous propofol in a dosage of 1–2.5 mg/kg body weight and fentanyl in a dosage of 1–1.5 mcg/kg of body weight.

When needed, RFA was guided by contrast-enhanced visualization of the lesion using standard ultrasound contrast medium (Sonovue, Bracco, Milano, Italy).

After prior lesion treatment by transarterial chemoembolization (TACE) or RFA, contrast medium-assisted evaluation was performed in order to identify residual tumor tissue.

The RFA system consisted of the Covidien Cool-tip ^TM^ RF Ablation System E Series and Covidien 17G Cool-Tip ^TM^ RF Ablation Single Electrode E Series needles (Medtronik, Watfort, UK). Needle characteristics (length, tip exposure creating radiofrequency) as well as ablation duration were individually chosen according to the target lesion characteristics. Lesions between 2 and 3 cm were approached with a 3 cm exposure needle and treated for 12 min. Lesions smaller than 2 cm were treated with a 2 cm exposure needle and ablation times up to 6 min.

The procedure was performed in the position that best allowed visualization and percutaneous approach to the hepatic lesion (supine or left lateral position). Prior to RFA needle placement, local anesthesia using 2% lidocaine was applied in every patient. Local anesthetics were injected in the subcutaneous tissue as well as into the Glissonean capsula. After lidocaine injection, the RFA needle was placed, and the patient sedated only afterwards in order to take advantage of the patient’s breathing manoeuvers. Once correct needle placement was ascertained by US, the anesthesiologist began with deep sedation using a propofol/fentanyl regime, with dosages as described above. Artificial ascites was created for lesions in a subcapsular location or close to other organ structures such as the diaphragm, colon or stomach using glucose 5% applied with a Verres needle (Medax Medical Devices, San Possidonio, Italy) under ultrasound guidance. Artificial ascites is needed in order to diminish or prevent organ damage/perforation to adjacent organs by thermoablation. The fluid creates a water cushion, generally absorbing heat during thermoablation.

**Figure 1:**
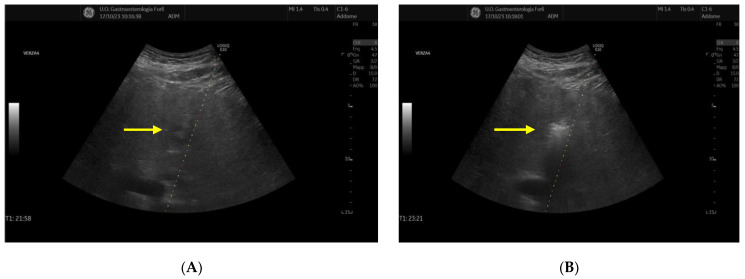
(**A**): Needle placement into an HCC nodule of the left liver lobe. (**B**): Thermoablation with RFA in the same patient. Yellow arrow indicates HCC nodule.

Then, current was applied and patients were maintained under deep sedation for the entire duration of RFA. Figure 1A shows RFA needle placement into an HCC of the left liver lobe and Figure 1B shows subsequent thermoablation.

All patients were hospitalized and peri-procedural antibiotic prophylaxis was given using a 3rd generation cephalosporin intravenously.

Pre- and postoperatively, white blood count and hepatic biochemistry results were controlled.

**Figure 2:**
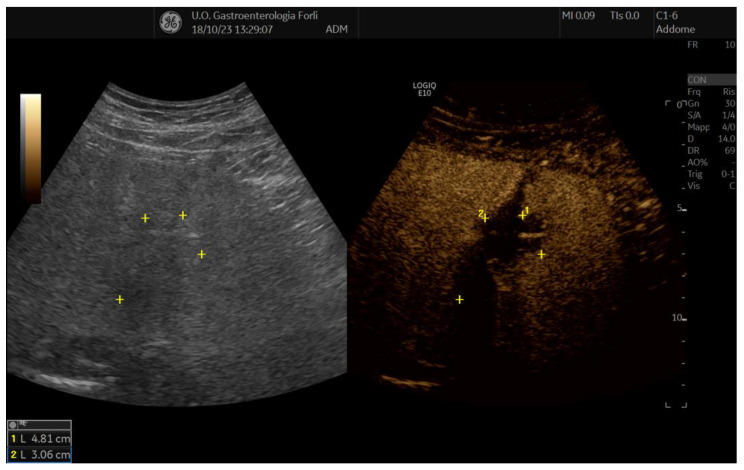
CEUS at day one post RFA showing a large ablation zone without contrast enhancement during the arterial phase. The ablated area remains avascular in the portal and late phase.

In order to assess early complications or residual tumor tissue, contrast-enhanced ultrasound (CEUS) examination was performed on postop day 1 (Figure 2).

**Figure 3:**
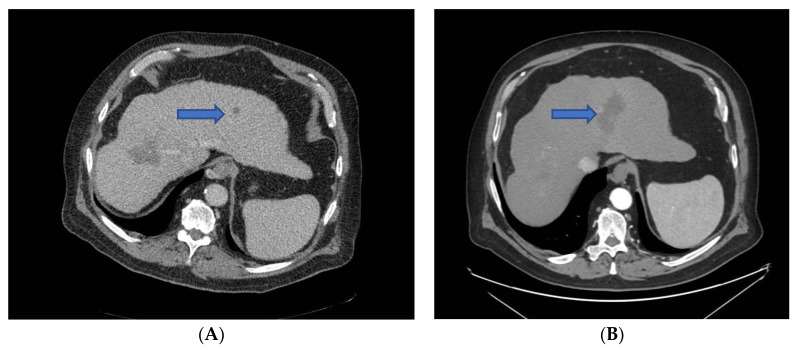
(**A**,**B**) show an example of a CT scan in a patient with a left lobar HCC nodule prior to (**A**) and at 1 month after (**B**) RFA (blue arrow indicating the HCC nodule).

A CT scan was routinely performed after 1 and 6 months for diagnosing early recurrence/residual tumor masses alongside the RFA zone (Figure 3A,B).

### 2.2. Outcomes

Primary outcomes were intraprocedural and post-procedural (within 30 days) RFA-related and anesthesia-related complications.

Hematoma, bleeding, abscess, bilioma, bile duct injury and pleural effusion accounted for RFA-related complications in the NORA setting. Hypotension, upper airway obstruction, hypertension and bradycardia accounted for immediate anesthesia-related complications.

Secondary outcomes were local recurrence rates at 1 month and 6 months after RFA. In order to detect early local recurrence, either CEUS or a CT scan was performed. Lesions adjacent to or deriving from the ablation zone with arterial hypervascularization and early- or late-phase washout were defined as local recurrence.

### 2.3. Data and Statistical Analysis

Data were collected in a prospectively maintained database.

Patient-related data including sex, age, body mass index (BMI), comorbidities, ongoing antiplatelet/anticoagulant therapy, tumor entity, tumor size and location were obtained. Data regarding etiology of liver disease, Child–Pugh score and MELD values, alpha-fetoprotein concentrations, and presence of portal vein thrombosis were also collected for patients who underwent RFA for HCC. Laboratory results prior to and after the procedure were collected. Postoperative data comprised hospital stay, CEUS at day one after the procedure, and CT scan after 1 and 6 months after RFA.

All data were analyzed retrospectively. Continuous variables are reported as mean ± standard deviation, and categorical variables are summarized as frequencies and percentages. Al statistical analyses were performed using Microsoft Excel (Software version 16.54).

## 3. Results

Between September 2021 and October 2023, a total of 35 patients were enrolled in this study and treated with RFA for various oncologic indications including 26 hepatocellular carcinomas, 6 colorectal liver metastases, and 3 metastases of gastric adenocarcinoma. Mean age was 71.5 years (SD 9.5) and 80% (28 patients) were male. Mean BMI was 29.1 (SD 5.2) and 34.3% of patients were obese. More than a half of patients presented with two or more comorbidities (mainly cardiologic and metabolic comorbidities). In addition, 37.1% of patients had a history of previous locoregional treatment (previous RFA in 20% and TACE in 2.9%) and surgical resection (14.2%) for HCC or metastasis.

Among patients undergoing RFA for HCC (n.26, 74.3%), the prevalent etiology of liver disease was Metabolic Dysfunction-Associated Steatotic Liver Disease. The majority of patients had CHILD PUGH A compensated liver cirrhosis (19 patients (73.1%) score 5, 4 patients (15.4%) score 6). Patient characteristics are displayed in Table 1.

Thirty-two patients (91.4%) had a single lesion. The median lesion’s size was 21 mm (SD 10.1 mm). Twenty-seven tumors (71.1%) were located in the right hepatic lobe. Four patients had subcapsular/subphrenic tumor locations. All procedures were performed under deep sedation, with patients receiving a propofol/fentanyl sedation in NORA. Procedural characteristics are shown in Table 2.

The RFA procedure completion rate was 100%, with a single needle insertion for each tumor nodule in order to achieve complete tumor ablation. No intraprocedural RFA-related complications (needle displacement, hematoma, gastrointestinal perforation, bile duct injuries) were observed. Only one post-procedural RFA-related complication was observed (2.86%). One morbidly obese patient (BMI > 40) presented with a large right-sided pleural effusion after undergoing RFA for two lesions next to the hepatic dome. He was treated conservatively with O_2_ therapy and furosemide i.v., leading to hospital dismissal on day 5. One patient developed post-RFA syndrome, showing fever without an infectious focus.

Abdominal ultrasound with an ultrasound contrast agent on postoperative day one demonstrated regular postoperative outcomes in all patients without signs of residual tumor tissue (necrotic avascular area on the site of the tumor nodule), intrahepatic hematoma or other complications associated with the procedure. The examination was always performed by the operator executing the RFA procedure.

No intraprocedural (hypotension, desaturation with necessity for intubation, inadequate pain control) and procedural anesthesia-related complications occurred.

Mean hospital stay was 3.5 ± 1.1 days. Re-hospitalization within 30 days and any other morbidity/mortality within 30 days after the intervention was not observed.

At 1 and 6 months after RFA, no tumor recurrence (HCC/CRLM) on the ablation site was detected. Therefore local recurrence-free survival was 100% at 6 months. In one elderly patient with an HCC nodule of 5 × 5 cm, a combination treatment of RFA and TACE was performed. Due to tumor size, RFA was meant for debulking and therefore not considered in the analysis for local recurrence-free survival. 

## 4. Discussion

Thermoablation is a minimally invasive treatment option that is widely used for hepatic tumors. Percutaneous RFA is one of the most common curative local ablation methods for HCC, due to its relative safety, low risk of complications and applicability; its use is established in BCLC guidelines. This technique is also a well-studied treatment method for metastatic liver tumors that are not candidates for surgical interventions due to impaired hepatic function, extrahepatic major comorbidities or difficult to reach surgically.

RFA is generally performed with local anesthesia combined with sedation. There are three painful stimuli/sources of pain during percutaneous ablation of liver lesions: initial skin puncture for local anesthesia, puncture of the Glissonian capsule and deeper pain associated with thermal tissue necrosis. Patients are more likely to experience severe pain in case of lesions localized in a subcapsular site, in the first liver segment or when the treated lesion is close to large vessels.

In our study, we adopted a step-up sedation approach, with local lidocaine applied subcutaneously and deeper to the Glissonean capsula. Needle placement was performed with the patient awake, to benefit from respiratory maneuvers, in some cases, enabling visualization of liver lesions during needle insertion. Once the needle was correctly placed, the anesthesiologist started with deep sedation, which was maintained for the entire duration of the ablation.

There is no consensus on the best approach for patient sedation in liver RFA. Anesthesiological, ultrasound or radiologic scientific societies do not discuss the importance of NORA in this specific setting. The best sedation and analgesia should alleviate procedural discomfort, reduce peri-procedural pain, ease the performance of the procedure and should not have a negative impact on oncological outcome. Sedation-related complications should be restricted to the minimum, taking into account patients’ comorbidities and sedation regime. Only small retrospective or randomized studies comparing different sedation regimes in RFA for liver tumors are to date available [17,18,20,21].

In our study, all 35 patients received a propofol/fentanyl sedation in a NORA setting, showing a complete ablation of the lesion in all patients without occurrence of RFA-related and anesthesia-related intraoperative or perioperative complications.

If post-procedural pain occurred, paracetamol or tramadol was administered, which always showed sufficient effect. No post-hospitalization/long-term pain medication was needed. Notably, more than half of our patients had a history of liver cirrhosis, with often alcohol and drug abuse. Moreover, one-third of all patients were obese (BMI > 30), more than 50% of all patients presented with cardiac or metabolic comorbidities and MASLD was the etiology of liver disease in almost 50% of our patients.

As known, patients with cirrhosis are often difficult to sedate due to altered hepatic function and previous history of alcohol and drug abuse. Also, BMI represents another factor that may highly affect procedural outcomes and may represent an issue for airway management. In these complex cases, it is crucial to have the best setting to safely perform RFA, with potential benefits both for peri-procedural and long-term oncological outcomes.

No intraprocedural RFA-related complications were noted in our cohort. Only one morbidly obese patient showed postoperative desaturation due to a right-sided pleural effusion that developed due to an inflammatory response after large ablations next to the diaphragm. This complication is known to exist and is reported to occur in 1.3% cases after RFA procedures [22]. Intraprocedural artificial ascites creation can augment pleural effusion. Song et al. report prolonged hospital stay when pleural effusion after RFA with artificial ascites developed [23]. In our case, the patient had two large ablation zones, one in the VIIIth and one in the VIth hepatic segment, and pleural effusion was interpreted as an inflammatory response. Conservative treatment led to complete resolution. In obese patients, the operator could have difficulty in placing needles percutaneously due not only to the increase of subcutaneous abdominal tissue but also to hepatic steatosis, which makes tumor visualization often difficult.

Post-radiofrequency ablation syndrome is a common phenomenon after RFA of solid liver tumors. Approximately one-third of patients undergoing percutaneous radiofrequency ablation of hepatic tumors develop delayed, transient flulike symptoms that can be treated conservatively and are significantly related to the volume of tissue ablated. Symptoms usually manifest 3 days after ablation and last 5 days [24].

As this is a well-described and frequent condition after liver RFA, we did not include symptoms like fever, malaise, chills, nausea or mild transaminase increase in the definition of RFA-related complications. In our cohort, a 5-day hospitalization was necessary for one patient because of prolonged fever, later interpreted as post-RFA syndrome as no infective focus was found.

Patients with lesions adjacent to the gallbladder or major vascular structures were excluded in our study. Although lesions adjacent to the gallbladder/within a range of <5mm to the bile duct or major vascular structures do not typically represent a contraindication, and complications such as transient cholecystitis or bile leaks are rare [22,23], we preferred to exclude these patients due to our initial experience in this method. Also, recent data suggest inferior oncologic outcomes in patients with peribiliary HCC treated with RFA [25]. The main liver RFA-related complications reported in the literature are tumor seeding (0.6%), infection/abscess (0.6%), hemorrhage (0.4%), hepatic infarction (0.2%), gastrointestinal perforation (0.1%) and mortality in 0.15% [22,25,26,27,28], Our data are in line with the frequency of complications reported in the literature. Specifically, we did not observe intraprocedural problems such as needle displacement or intrahepatic hematoma.

To date, no studies specifically investigating anesthesia-related complications in liver RFA exist. Nevertheless, adverse effects of sedation in NORA procedures have been reported. Over the years, the interest in NORA has been rising, together with the increase in patients’ comorbidity, widespread mini-invasive procedures and a culture of pain-free procedures, with the aim of increasing patients’ comfort. Propofol-based sedation regimens are frequently applied by anesthesiologists in our unit, mostly for GI procedures. Current GI guidelines recommend propofol sedations rather than traditional sedations using midazolam due to better sedation, patient cooperation, the higher quality of the endoscopic procedure, and higher post-procedural patient satisfaction. It decreases time to sedation, recovery and discharge times. Guidelines stress that propofol regimens are even recommended in highly comorbid patients presenting with ASA 3 or Mallampati 3 or higher [19,20].

A large registry study conducted by Chang et al. analyzed effects and complications of NORA in different settings. In 12,252,846 patients receiving NORA for various reasons, minor complications occurred in 1.06% with nausea and vomiting, 1.01% inadequate pain control and 0.62% minor hemodynamic instability. Major complications comprised upgrade of care in 0.1% and severe hemodynamic instability in 0.1% [26]. Our data are in line with the frequency of complications reported in this large observational study. We did not observe hemodynamic instability, hypotension or a need for intubation.

To date, there are only few studies comparing different sedation regimens for liver RFA that focus on pain, patient satisfaction or hospitalization.

Previous studies have documented that over 40% of patients under conscious sedation reported severe intensity of pain during RFA [29,30,31]. Conscious sedation usually consists of a combination of benzodiazepine/analgesics. However, literature data show how this sedation regime is often inadequate.

In a randomized trial with 120 patients, Wu et al. compared two sedation regimes, one group using dexmedetomidine with oxycodone (single i.v. injection) and the other group using dexmedetomidine with remifentanil (continuous infusion). When a combination of dexmedetomidine and oxycodone was used, patients had significantly less peri-procedural pain, required fewer analgesics in the first 24 h after RFA and had higher patient satisfaction [27].

In a recent review, Piccioni et al. discuss different sedation regimes for RFA including midazolam/propofol/remifentanil–fentanyl, total paravertebral blocks and thoracic epidural anesthesia. Data concerning different sedation approaches come from fairly small studies that mostly are not randomized/controlled. Piccioni et al. conclude that sedation regimes should be chosen upon local expertise as no representative data are available. They conclude that general anesthesia should be avoided when performing liver RFA, as it may lead to slower recovery and obviously does not enable the patient to cooperate during liver RFA [19].

Long-term anesthesiological complications are studied even less in the setting of RFA. Importantly, the sedation regimen could have an impact on oncological short- and long-term outcomes, when patient sedation is not sufficient and intraoperative needle displacement occurs. A recent study by Puijk et al. compared the use of general anesthesia, propofol and midazolam/fentanyl-based sedation regimes in thermoablation (microwave ablation for HCC and CRLM). They found that patients treated with midazolam/fentanyl-based sedation had significantly higher peri-procedural perceptions of pain and, interestingly, higher local tumor recurrence rates compared with patients receiving either propofol or general anesthesia [18].

When considering oncological outcomes, in our cohort complete tumor ablation was achieved in 100% of patients within the first RFA session, and no residue/recurrence was observed at 1 and 6 months (local tumor progression-free survival of 100%).

These results are in line with evidence emerging from a previous study by Kuo et al. investigating oncological short-term and long-term outcomes of RFA. Indeed, patients treated with general anesthesia needed fewer interventions for complete tumor ablation (92.3% complete tumor ablation after one RFA procedure in patients with general anesthesia compared with 92.4% complete tumor ablation after 1–3 RFA procedures in patients without general anesthesia). Nevertheless, long-term oncological outcomes were not different between both groups [28].

These data highlight how deep sedation leads to a more precise and controlled ablation, improving the possibility of achieving complete ablation zones and therefore sufficient oncologic outcomes.

The present study has several limitations: first, its retrospective design leads to numerous biases; second, the small cohort size and the absence of a control group; and, third, the lack of long-term oncological outcomes. Further large cohort studies comparing different sedation regimens for thermoablation are necessary in order to confirm our preliminary data showing good outcome of deep sedation. Longer follow-up of our cohort is necessary to compare local recurrence-free survival rates with those reported in the literature. Therefore, future research including larger cohorts, different sedation regimes and longer oncological follow-up are warranted.

## 5. Conclusions

RFA for HCC and CRLM is a valid treatment alternative to surgery in well-defined clinical situations. Deep sedation for RFA may play a crucial role, not only for patient and operator comfort during the procedure but also for long-term oncologic outcomes. Our study demonstrates a positive effect on RFA outcomes when deeper sedation protocols are used. However, larger and foremost comparative studies are needed to confirm this effect. The positive effects of NORA seen in our study may not automatically be present when applied for other interventions and have to be investigated separately.

## Figures and Tables

**Table 1:** Patient characteristics.

	Total pts (*n* = 35)	HCC (*n* = 26)
Age, years (mean (SD))	71.5 (9.5)	70.8 (8.3)
Gender, male (*n* (%))	28 (80)	21 (80.8)
BMI (mean (SD))	28.5 (5.2)	29.1 (5.2)
HCC (*n* (%))	26 (74.3)	
Metastasis (*n* (%))	9 (25.7)	
Colon cancer (*n* (%))	6 (66.7)	
Gastric cancer (*n* (%))	3 (33.3)	
Comorbidities		
Obesity (*n* (%))	12 (34.3)	12 (46.1)
Ischemic heart disease (*n* (%))	6 (17.1)	5 (19.2)
Hypertension (*n* (%))	16 (45.7)	13 (50)
Dyslipidemia (*n* (%))	3 (8.5)	3 (11.5)
Diabetes mellitus (*n* (%))	11 (31.4)	9 (34.6)
COPD/asthma (*n* (%))	4 (11.4)	3 (11.5)
Atrial fibrillation (*n* (%))	4 (11.4)	4 (15.4)
Previous treatment (*n* (%))	13 (37.1)	
Liver resection (*n* (%))	5 (38.5)	
RFA (*n* (%))	7 (53.8)	
TACE (*n* (%))	1 (7.7)	
Antiplatelet therapy (*n* (%))	7 (20%)	
Anticoagulant therapy (*n* (%))	6 (17.1)	
Vitamin K antagonists (*n* (%))	2 (33.3)	
DOACs (*n* (%))	2 (33.3)	
Heparins (*n* (%))	2 (33.3)	
Etiology of liver disease		
ALD (*n* (%))		6 (23.1)
MASLD (*n* (%))		12 (46.1)
AIH (*n* (%))		1 (3.8)
PBC (*n* (%))		1 (3.8)
PSC (*n* (%))		0 (0)
HBV infection (*n* (%))		4 (15.4)
HCV infection (*n* (%))		9 (34.6)
Cryptogenic (*n* (%))		1 (3.8)
Mixed etiology (*n* (%))		9 (34.6)
Chronic liver disease/cirrhosis (*n* (%))		24 (92.3)
Child–Pugh score		
A5 (*n* (%))		19 (73.1)
A6 (*n* (%))		4 (15.4)
B7 (*n* (%))		2 (7.7)
B8 (*n* (%))		1 (3.8)
MELD-Na (mean (SD))		9.2 (2.4)
Alpha-FP value, ng/mL (mean (SD))		12 (14.5)
Portal vein thrombosis (*n* (%))		2 (7.7)

Abbreviations: BMI, body mass index (kg/m2); HCC, hepatocellular carcinoma; COPD, Chronic obstructive pulmonary disease; RFA, radiofrequency ablation; TACE, Transarterial Chemo-Embolization; DOACs, direct-acting oral anticoagulants; ALD, Alcohol-related liver disease; MASLD, Metabolic Dysfunction-Associated Steatotic Liver Disease; AIH, autoimmune hepatitis; PBC, primary biliary cholangitis; PSC, primary sclerosing cholangitis; Alpha-FP, alpha-fetoprotein. HBV, Hepatitis B Virus; HCV, Hepatitis C Virus.

**Table 2:** Procedural features (pts *n*. 35, nodules *n*. 38).

RFA	
Tumor number	
1 (*n* (%))	32 (91.4)
2 (*n* (%))	3 (8.6)
Tumor location	
S1 (*n* (%))	0 (0)
S2 (*n* (%))	1 (2.6)
S3 (*n* (%))	4 (10.5)
S4 (*n* (%))	6 (15.8)
S5 (*n* (%))	3 (7.9)
S6 (*n* (%))	9 (23.7)
S7 (*n* (%))	9 (23.7)
S8 (*n* (%))	6 (15.8)
Tumor size, mm (mean (SD))	21 (10.1)
Size of needle’s tip	
2 cm (*n*, %)	6 (15.8)
3 cm (*n*, %)	32 (84.2)
Artificial ascites—subcapsular or subphrenic lesions (*n* (%))	4 (10.5)
Duration of ablation time, min (mean (SD))	8.8 (3.1)
Peak power, W (mean (SD))	152.7 (22.2)
Anesthesia	
Sedation agents	
Propofol + fentanyl (*n* (%))	35 (100%)
Insertion of the needle with the patient awake (*n* (%))	35 (92.1)

Abbreviations: RFA, radiofrequency ablation; W, Watt.

## Data Availability

Data is available on request due to restrictions (privacy reasons).

## Abbreviations

ABCDE	Airway, Breathing, Circulation, Disability, Environment
BCLC	Barcelona Clinic Liver Cancer
CRLM	Colorectal liver metastases
CT	Computed tomography
ECOG	Eastern Cooperative Oncologic Group Performance Status
ESMO	European Society for Medical Oncology
HCC	Hepatocellular carcinoma
i.v.	Intravenous
MASLD	Metabolic-Dysfunction Associated Steatotic Liver Disease
MWA	Microwave ablation
NORA	Non-operating room anesthesia
RFA	Radiofrequency ablation
TACE	Transarterial chemoembolization
US	Ultrasound
